# Transforming aggression into creativity: Creative thinking training as a new strategy for aggression intervention

**DOI:** 10.1002/pchj.713

**Published:** 2023-12-17

**Authors:** Jiaqi Wu, Yongqiang Yang, Xiaofei Wu, Ziyi Li, Jing Luo

**Affiliations:** ^1^ Beijing Key Laboratory of Learning and Cognition, School of Psychology Capital Normal University Beijing China; ^2^ Department of Psychology Shaoxing University Shaoxing China

**Keywords:** aggression, creative thinking training, creativity, intervention

## Abstract

Although reducing students' aggressive behaviors and improving their creativity are both important commitments of educators, they are usually treated independently as unrelated tasks. However, cumulative evidence suggests potential links between aggression and creativity, not only from the perspectives of personality traits and psychological development, but also from their shared cognitive mechanisms. This implies that there may be an approach to achieving these two goals through a single intervention. Moreover, this new approach may overcome the limitation of the usually adopted aggression intervention, which is limited in its regulatory effectiveness and might bring about some disadvantageous impacts on creativity that are closely associated with aggression. To test this possibility, the present study implemented a four‐session, 2‐week creative thinking training (CTT) intervention for students with high aggression scores to examine whether it could simultaneously downregulate aggression and increase creativity. Our results demonstrate that, compared to the control group, the intervention group experienced significant improvements in creativity and a reduction in aggression following the CTT intervention. Furthermore, our findings suggest that this regulatory effect can persist for up to 6 months. The CTT‐induced creativity change (increase) could significantly correlate with and predict the CTT‐induced aggression decrease, thus suggesting that the CTT could transform aggression into creativity.

## INTRODUCTION

Some negative traits may boost creativity (Fink et al., 2014; Greenwood, 2020). Ernest Hemingway, known for his adventurous spirit and spare direct style of writing, was involved in several fights and was expelled from high school for breaking the rules. Although his aggressive tendencies may have caused him problems at the time, they also contributed to his intense and passionate personality, which he channeled into his creative writing (Baker, 1969). Additionally, in the work *The Naughty Boys at the Crossroads*, Wang (2006) wrote over 100 cases of famous scientists and innovators, including Nobel Prize winners, who had experienced a mischievous or even aggressive childhood but finally made outstanding scientific creative achievements after a psychological transformation.

Aggression is the desire and behavior of an individual to intentionally harm others (Anderson & Bushman, 2002), whereas creativity is the generation of novel things that are useful and appropriate (Benedek et al., 2023). Despite the fact that reducing aggressive behaviors and improving creativity are both important commitments to education (Rafi et al., 2020; Wagner, 2019), these two commitments are usually treated independently as unrelated affairs. However, cumulative evidence has revealed a potential link between aggression and creativity.

### Aggression and creativity

It has been suggested that aggression and creativity share a common origin (Tacher & Readdick, 2006). Rebelliousness and rule‐breaking were found to boost creativity at an appropriate level (Petrou et al., 2018, 2020), and verbal duels, which are usually a cathartic form of aggression, could also involve creativity and artistic value (Pagliai, 2009; Progovac & Benítez‐Burraco, 2019). Moreover, from the perspective of individual development, second‐grade students create and display uncommon or unique gestural and verbal aggressive behaviors (e.g., threats) instead of physical aggression to stop others' unwanted behaviors (Tacher & Readdick, 2006) and these could also occur as a kind of developmental adaptation with creative features (Lazarus, 1991). A study of the developmental trajectory of creativity in 3rd‐ and 4th‐grade children found that those who showed increased creativity also showed increased rule‐breaking behaviors and aggression (Saggar et al., 2019). Consistently, a qualitative study involving teachers' impressions and beliefs about highly creative student characteristics suggested that these students may have traits of impulsivity, arrogance, dominance, rule‐breaking, and rebelliousness (Gralewski, 2019), many of which are also associated with aggression (Volk et al., 2022). Results from a meta‐analysis showed that impulsivity not only leads to violent aggression (Rogier et al., 2019), but also promotes unrestrained expressiveness and fosters creativity in appropriate circumstances (Greenwood, 2020). In addition, individuals who are rebellious tend to break rules and produce creative ideas (Petrou et al., 2018). Studies on the Big Five personality have found that neuroticism can positively predict both aggression and creativity (Gao et al., 2020; Hu et al., 2022), and psychoticism, a personality trait (e.g., cold, unempathetic, aggressive, and impulsive behaviors) is considered to contribute positively to the originality of creativity (Fink et al., 2014).

### Anger and creativity

In addition, aggression may improve creativity by inducing individuals to think outside the box and find uncommon ways to create novel ideas (Hennessey & Amabile, 2010). Rule‐breaking behaviors can boost creativity especially in a context of constraints (Petrou et al., 2018). Anger, which is always associated with aggressive traits (Anestis et al., 2009), was also found to promote creative performance by making individuals feel less restricted by the ordinary way of thinking and more focused on novel problem‐solving approaches (Zhan et al., 2020). It is consistent with previous studies involving negative moods promoting problem‐solving (Shen et al., 2019). Moreover, anger can also enhance individuals' cognitive activation level and enrich more cognitive resources to invest in the current task (Cheng et al., 2021), which could be especially helpful for creation. This reflects an important feature of the task of divergent thinking: people need to break common associations under inflexible rules and construct new associations between previously remote elements (Guilford, 1950). That could be achieved through the aggression and anger that mobilize intensive psychological energy and weaken the top‐down control function of the prefrontal cortex, which essentially subserves the ordinary way of thinking and rule obeying.

The link between aggression and creativity may have important implications for interventions. First, it questions whether the usual approaches of inhibition (Romero‐López et al., 2021) and self‐control training (Beames et al., 2023) to control and reduce aggression may also be less beneficial to the valuable capability of creativity. Second, and more importantly, it suggests that we may be able to find a way to improve creativity while simultaneously reducing aggression. Therefore, rather than suppressing and hiding aggression, we need to consider how to constructively use aggression to fulfill individual needs within the boundaries of society (Pool & Odell‐Miller, 2011) and transform it into creativity.

### Interventions for aggression and creativity

In recent years, the intervention approach has reduced aggression by promoting internal self‐control ability (Ridge et al., 2023; Romero‐López et al., 2021). However, interventions that rely solely on improving self‐control to reduce aggression are not as effective, as their impact on aggression involves a far transfer through an indirect capacity improvement, rather than a direct target to aggression itself (Beames et al., 2023). Views on psychological treatment suggest that aggression is something we must learn to live with and that we need to enable aggressive expression when aggression is suppressed inappropriately (Pool & Odell‐Miller, 2011). To some extent, aggression involves biological adaptations to overpower an opponent and achieve a certain goal (Faris et al., 2020; Lischinsky & Lin, 2020). However, owing to the restrictions and rejection of aggression by society, individuals adaptively produce non‐aggressive alternative behaviors to achieve their own goals (Progovac & Benítez‐Burraco, 2019; Saggar et al., 2019; Tacher & Readdick, 2006). Anderson and Bushman (2002) suggested that if a person is not satisfied with the current solution and has sufficient time and cognitive resources, impulsive action (i.e., aggression) is replaced by thoughtful action (i.e., creativity) after reappraisal, during which information is repeatedly considered and judged. This process is similar to creative cognition, which requires evaluating the usefulness of the generated ideas and continuously filtering them to meet specific goals (Beaty et al., 2016). Some studies of art interventions have implications for the transformation of aggression into creativity. A case study of music therapy found that after music therapy, patients' self‐confidence in mastery of impulsivity and aggression increased, suppressed emotions were expressed constructively, and their intention sublimated from aggression to music creation (Pool & Odell‐Miller, 2011). Moreover, art interventions, such as drama, music, and dance, have also demonstrated the effectiveness of aggression reduction and improving artistic creation in prinsoners (Grigorenko, 2020). Therefore, this study aimed to provide positive guidance rather than one‐sided suppression by reshaping and developing the potential positive aspects of aggression (Holm‐Hadulla, 2020). Specifically, this study attempted to transform the aggression of more aggressive students into creativity through creative thinking training (CTT), thereby achieving the goal of promoting creativity and suppressing aggression.

### The present study

An early meta‐analysis study pointed out that creativity can be significantly improved through training and that adequate training programs will pay more attention to developing cognitive skills and practical practice in reality (Scott et al., 2004). In the present study, we examined the “double” effects of CTT on the promotion of creativity and inhibition of aggression. CTT can enhance creativity (Kienitz et al., 2014). For example, some process‐oriented training methods were utilized to train students to master certain cognitive direction techniques to comprehensively enhance their creativity (Gu et al., 2022; Ritter & Mostert, 2017). Specifically, Ritter and Mostert (2017) conducted creativity training (involving silence, lines of evolution, random connections, and SCAMPER) and discovered improvements in divergent thinking, convergent thinking, and creative problem‐solving abilities. This means that through training, performance on creative tasks can be improved. Moreover, in the field of cognitive neuroscience, cognitive training has been found to change brain structures. Fink et al. (2010) used classic group‐based brainstorming techniques (Osborn, 1957) to test whether creativity can be improved during the process of idea sharing and idea exchange through the intervention of exposure to external (or other people's) ideas in fMRI. They found that this intervention was effective in improving originality and that this performance improvement was associated with increased activation of a neural network in the right hemispheric temporoparietal, medial frontal, and posterior cingulate cortices bilaterally. A 1‐month cognitive stimulation training task enhanced participants' divergent thinking ability, and the results showed that functional changes occurred in brain regions such as the posterior cingulate gyrus, dorsolateral prefrontal cortex (PFC), and inferior parietal lobule after training, while the brain regions that underwent structural changes owing to training were mainly the posterior cingulate gyrus (Sun et al., 2016). These changes in brain areas indicate that after CTT, individuals' cognitive control and ability to generate novel connections are improved. On the other hand, CTT may also reduce students' aggressive behavior by facilitating the activation of the PFC, which could improve cognitive control over aggression. Raine et al. (2000) discovered that individuals exhibiting antisocial personality disorders and violent tendencies showed smaller PFC volumes, hindering their ability to regulate anger. Following creative training, there is an observable increase in resting‐state functional connectivity between the medial PFC and medial temporal gyrus (Wei et al., 2014). Additionally, training programs have demonstrated that enhanced creativity coincides with improvements in executive function (Zhao et al., 2023). Aggressive behavior is primarily linked to compromised executive function (Giancola, 2004). Therefore, CTT not only aids in the development of creativity but also has the potential to alleviate or redirect aggression.

In summary, this study developed a CTT program for aggression intervention and creativity promotion. We trained students with high aggression traits through a series of training sessions and tested whether this intervention could reduce aggression and prompt creativity. The present study proposes the following hypotheses: (1) there is a positive correlation between aggression and creativity; (2) participants who receive intervention in CTT exhibit a more significant reduction in aggression and enhancement in creativity compared to the control group; (3) the decrease in aggression can positively predict the increment in creativity; and (4) the intervention effect persists for 6 months after the completion of CTT.

## METHOD

### Participants

A total of 189 students (134 males, *M*_age_ = 17.12 years; 55 females, *M*_age_ = 17.18 years) were recruited from a high school in Shanxi Province, China to participate in the present experiment. The research protocol was approved by the Ethics Committee of Capital Normal University, and permission was obtained from the school principal to conduct the study. Following a screening process involving questionnaires, participants who scored within the top 33% in terms of aggression (with the screening criteria kept confidential from the students) were invited to participate voluntarily in subsequent intervention experiments. Additionally, before the training, participants were required to provide informed consent and obtain consent from their legal guardians. Ultimately, 62 students (44 males, *M*_age_ = 17.00 years; 18 females, *M*_age_ = 17.17 years) participated in the training program (after randomization, 32 in the intervention group, 30 in the control group).

### Measures

#### Buss and Perry Aggression Questionnaire

Aggression levels were assessed using the Buss and Perry Aggression Questionnaire (BPAQ; Buss & Perry, 1992). The participants completed a revised Chinese version of the questionnaire that has been tested for reliability and validity (Li et al., 2011). The questionnaire consists of 29 items categorized into four subdimensions: physical aggression, verbal aggression, anger, and hostility. The scores are rated on a 5‐point Likert scale ranging from 1 (*never*) to 5 (*always*). Higher overall scores indicate a greater level of aggression. In this study, Cronbach's alpha was .860.

#### Williams Creativity Scale

The Williams Creativity Scale (WCS), originally developed by Williams (1979) and revised by Liu et al. (2016), was used to evaluate creative traits. The subdimensions include curiosity, imagination, challenge, and risk‐taking. The questionnaire consists of 50 items. Participants rated each item on a 3‐point Likert scale (total score of 150 points), and seven items were reverse‐scored, with higher scores reflecting greater levels of creativity. The total scale exhibited excellent internal consistency within the current sample (Cronbach's alpha = .878).

#### Torrance Tests of Creative Thinking

The Torrance Tests of Creative Thinking (TTCT) are the most widely used measures of creativity (Torrance, 1972, 1974). There are two versions of the TTCT: the TTCT‐Verbal and the TTCT‐Figural. In this study, we used the TTCT‐Figural, which is better suited for measuring creativity (Kim, 2017). Participants were instructed to complete the TTCT‐Figural Form A (lines) test, create drawings, and provide titles or explanations for their drawings. After completing the TTCT‐Figural, scores were assigned based on three dimensions (fluency, flexibility, and novelty) (Teng et al., 2022). The fluency was measured based on the number of completed drawings. Flexibility was scored based on the number of different response categories. Novelty was rated based on the relative frequency of each unique response across all drawings produced in the entire experiment on a scale ranging from 1 to 5 (Runco et al., 2016).

### Procedure

**FIGURE 1:**
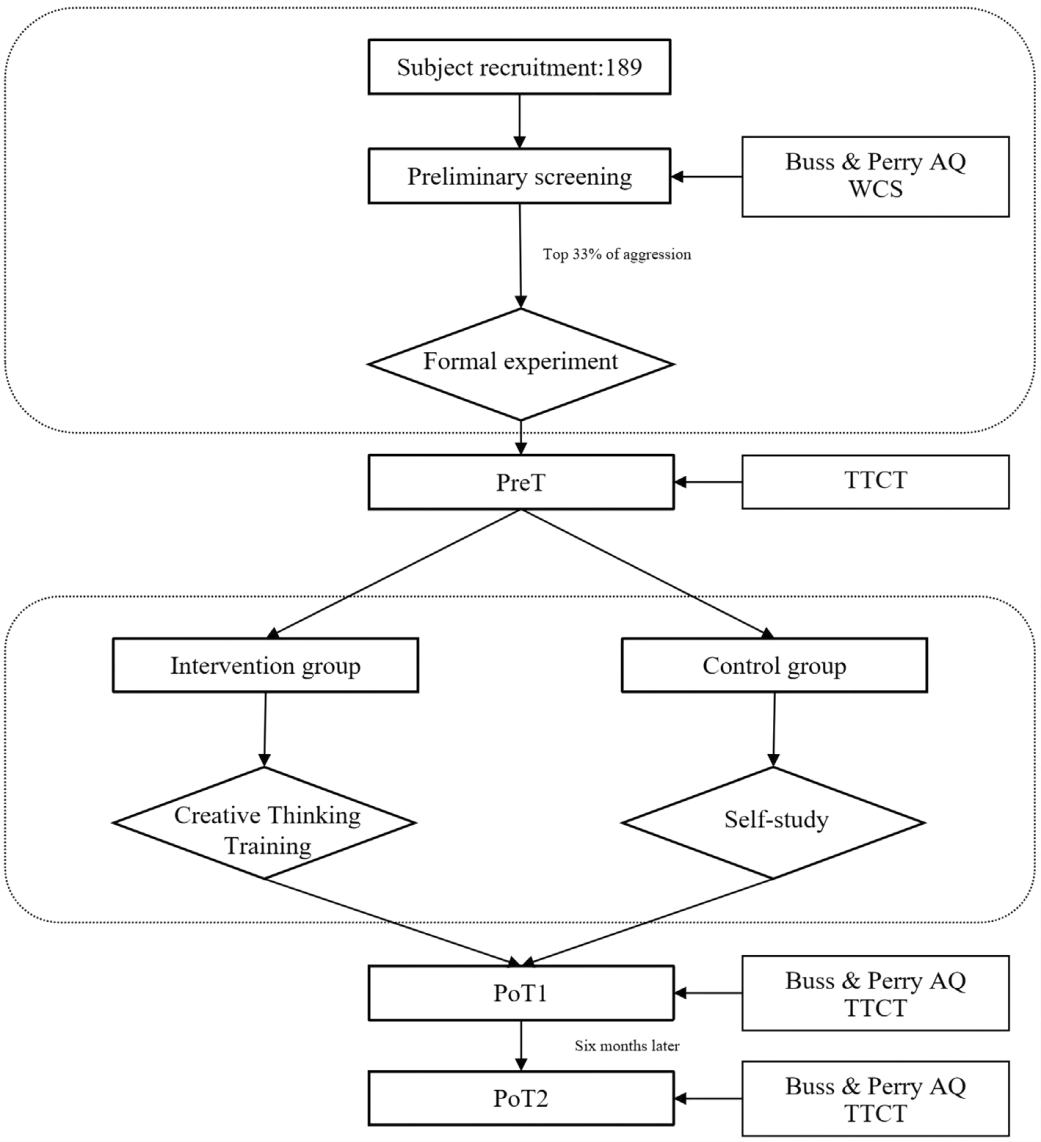
Experimental flow graph.

The experiment consisted of five phases: participants were screened to select those with relatively high aggression traits, pre‐test of aggressive and creative scores (abbreviated as PreT), intervention of CTT, post‐test of aggressive and creative scores (post‐test 1 [PoT1]), and a 6‐month follow‐up to track the effectiveness of the intervention (post‐test 2 [PoT2]) (see Figure 1).

#### Screening phase

All participants were administered the BPAQ and WCS. Responses with a completion time larger than ±3 standard deviations and those with missing answers were excluded, resulting in a final sample of 172 participants (out of 189 individuals, 90.5%). From this sample, students with the top 33% of aggression scores (including those with repeated scores) were selected, resulting in 64 more aggressive participants with a BPAQ score of 3.09 ± 0.30 (46 males, 18 females), who were randomly assigned to an intervention group of 32 participants (21 males) and a control group of 32 participants (25 males). The participants in the intervention group and the control group were conducted in separated classes. These 62 selected participants (32 in the intervention group, 30 in the control group, and two male participants who withdrew from the experiment owing to academic obligations) voluntarily signed an informed consent form approved by the school, teachers, and local legal guardians. Participants underwent pre‐testing, intervention, post‐testing, and follow‐up testing.

#### PreT phase

All participants completed the TTCT‐Figural. The tests were administered in a quiet classroom setting, with the examiner reading the instructions and two schoolteachers maintaining the testing environment. The total testing time was 30 min.

#### Intervention phase

**FIGURE 2:**
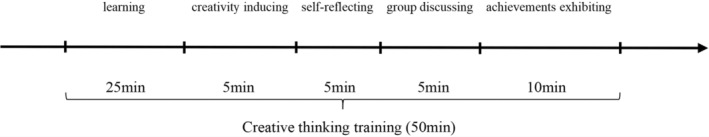
Flowchart of a single intervention experiment.

The intervention group received CTT in the classroom, whereas the control group engaged in self‐study. The intervention training lasted for 2 weeks, with two sessions per week, and each session lasted approximately 50 min (this is the length of one class), totaling four CTT sessions. In each intervention session, CTT included five sub‐stages (learning, creativity induction, self‐reflection, group discussion, and achievements). In the learning stage of CTT, the experimenter first provided instruction and the task of intervention training (the training content is described in the following paragraphs); then, the experimenter provided students with some creative inspiration (watching creative videos, showcasing creative products, and displaying creative images) to encourage creativity. Students were also required to reflect on creative thinking. Finally, students randomly formed groups of four to discuss and generate their own creative ideas and exhibited the group's representative achievements to the entire class (see Figure 2).

#### Contents of CTT

Four types of training games or tasks for promoting creative thinking were assigned in the four training sessions (one task per session): (1) The task of *combination* involves intentionally merging two or more seemingly unrelated things together to create a new thing, solve a problem, or create a story (M. Sun et al., 2020). For example, participants were asked to use the three words “limping dog, police, bicycle” to create an interesting and unique story. (2) The task of *transformational thinking* requires individuals to solve a problem from a completely new perspective when the initial way of thinking fails to work. Based on the commonly used SCAMPER rule (Serrat, 2017), which stands for “substitute, combine, adapt, modify, put to other use, eliminate, and rearrange,” we designed cognitive tasks on how to generate a new “Frappuccino.” Crushed peanuts can be substituted with jam, cream can be combined with biscuits, the ratio of raw materials can be adapted, a handle can be modified on the cup, co‐branded packaging can be used for collection, ingredients can be eliminated according to consumer preferences to make customized models, and hot stars can be rearranged into a Frappuccino. (3) The task of *principle‐based* thinking starts with a specific method to solve a problem and then requires participants to analyze the underlying principle and use it to expand their thinking and generate more alternative solutions (Ritter & Mostert, 2017). For example, participants were instructed to use the strategy of thinking about universal principles to solve the problem of preventing mosquito bites in the summer. In our daily lives, we wear clothes to keep mosquitoes away. The universal principle is to establish an “isolated” relationship. Therefore, we used fine gauze to create mosquito nets or compounds that mosquitoes dislike to produce mosquito‐repellent. (4) *Reverse thinking* encourages thinking outside the box and breaks conventional thinking when it fails (Sak & Oz, 2010). For example, to improve the design of cars, reverse thinking in daily life includes unmanned vehicles, which directly negate conventional thinking (cars do not need to be driven by humans), cable car cars belong to subject–object reversal (car body is under the wheel), and shared rental cars break the tradition (people do not need to buy cars). Each training session used one of four training types. It should be noted that these trainings were conducted in the classroom, and we did not record and evaluate each participant's performance in each session of creative training; we only estimated the holistic effects of four training sessions by assigning the final, post‐training evaluation (PoT1 and PoT2) and contrasting it with the pre‐testing scores (PreT).

#### PoT1 phase

The intervention and control groups completed the BPAQ and TTCT‐Figural immediately after completing all training (non‐training, control) sessions. All tests were administered in a quiet classroom setting, with the researcher serving as the test administrator and providing instructions. Two schoolteachers maintained their testing environments. The total testing time was 30 min.

#### PoT2 phase

After 6 months, the BPAQ and TTCT‐Figural were again administered to 31 participants in the intervention group (one participant was lost due to academic obligations).

## RESULTS

### Data collection difference test

Independent‐samples *t*‐tests found no significant between‐group differences in age, aggression, subdimensions of aggression, creative traits, or TTCT‐Figural scores (Table 1).

**TABLE 1:** Difference test between intervention group (21 males) and control group (25 males) in pre‐test.

	*M* ± SD (Group 1)	*M* ± SD (Group 2)	*t*	Cohen's *d*
Age (years)	17.03 ± 0.59	17.07 ± 0.58	−0.24	−0.01
Aggression	3.12 ± 0.31	3.04 ± 0.28	1.01	0.25
Physical aggression	2.64 ± 0.56	2.70 ± 0.48	−0.45	0.12
Verbal aggression	3.33 ± 0.49	3.17 ± 0.49	1.23	0.33
Anger	3.08 ± 0.57	3.07 ± 0.45	0.11	0.02
Hostility	3.51 ± 0.59	3.33 ± 0.57	1.18	0.31
Creative traits	2.17 ± 0.25	2.12 ± 0.15	0.89	0.24
TTCT‐Figural	10.94 ± 4.79	10.43 ± 5.46	0.39	0.01

*Note*: Group 1, intervention group, *N* = 32; Group 2, control group, *N* = 30.

Abbreviations: *M*, mean; SD, standard deviation; TTCT, Torrance Tests of Creative Thinking.

### Effect of intervention

**FIGURE 3:**
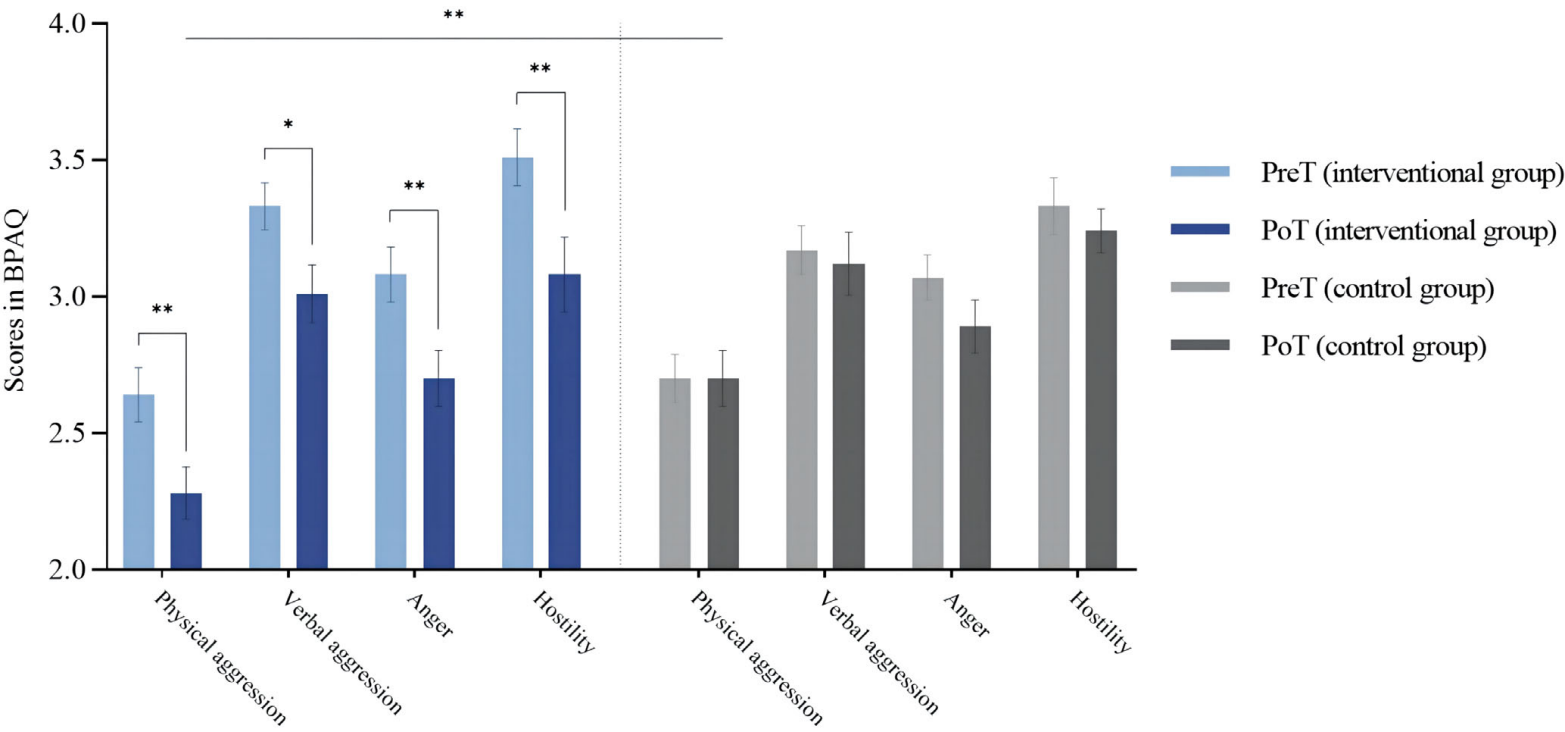
Subdimensions of aggression in the pre‐test and post‐test. BPAQ, Buss and Perry Aggression Questionnaire.

Independent‐samples *t*‐tests were conducted for the intervention and control groups after CTT (Table 2) to examine whether there were significant between‐group differences in aggression, subdimensions of aggression and creativity. The results showed that the intervention group had significantly lower levels of aggression and physical aggression, and significantly higher levels of creativity than the control group (Table 2; Figure 3).

**TABLE 2:** Difference test between intervention group and control group in post‐test.

	*M* ± SD (Group 1)	*M* ± SD (Group 2)	*t*	Cohen's *d*
Aggression	2.73 ± 0.43	2.97 ± 0.36	−2.39*	0.61
Physical aggression	2.28 ± 0.54	2.70 ± 0.56	−3.02**	0.76
Verbal aggression	3.01 ± 0.60	3.12 ± 0.63	−0.74	0.18
Anger	2.70 ± 0.58	2.89 ± 0.53	−1.37	0.34
Hostility	3.08 ± 0.78	3.24 ± 0.44	−0.97	0.25
TTCT‐Figural	19.82 ± 6.46	12.50 ± 4.40	5.17***	1.32

*Note*: Group 1 = intervention group, *N* = 32; Group 2 = control group, *N* = 30.

Abbreviations: *M*, mean; SD, standard deviation; TTCT, Torrance Tests of Creative Thinking.

* *p* < .05;

** *p* < .01;

*** *p* < .001.

**FIGURE 4:**
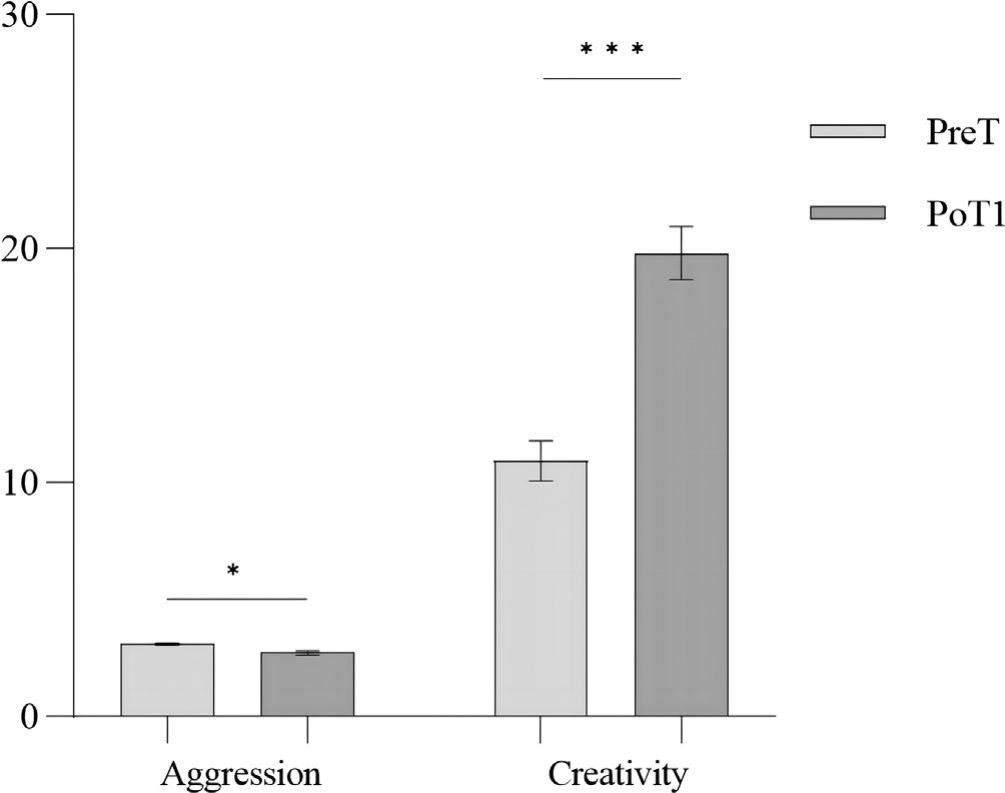
Variables in the pre‐test and post‐test for the intervention group.

Subsequently, a paired‐sample *t*‐test (Figure 4) was conducted to compare the PreT and PoT1 levels of aggression, subdimensions of aggression and creativity in the intervention group. The results indicated a significant difference (decrease) in aggression and subdimensions of aggression between the PreT and PoT1. Similarly, there was a significant difference (increase) in creativity between the PreT and PoT1 (Table 3).

**TABLE 3:** Difference test between pre‐test and post‐test in the intervention group.

	*M* ± SD (pre‐test)	*M* ± SD (post‐test)	*t*	Cohen's *d*
Aggression	3.12 ± 0.31	2.73 ± 0.43	4.47***	1.04
Physical aggression	2.64 ± 0.56	2.28 ± 0.54	3.36**	0.65
Verbal aggression	3.33 ± 0.49	3.01 ± 0.60	2.47*	0.58
Anger	3.08 ± 0.57	2.70 ± 0.58	3.73**	0.66
Hostility	3.51 ± 0.59	3.08 ± 0.78	2.91**	0.62
TTCT‐Figural	10.94 ± 4.79	19.82 ± 6.46	−9.56***	1.50

*Note*: *N* = 32.

Abbreviations: *M*, mean, SD, standard deviation; TTCT, Torrance Tests of Creative Thinking.

* *p* < .05;

** *p* < .01;

*** *p* < .001.

**FIGURE 5:**
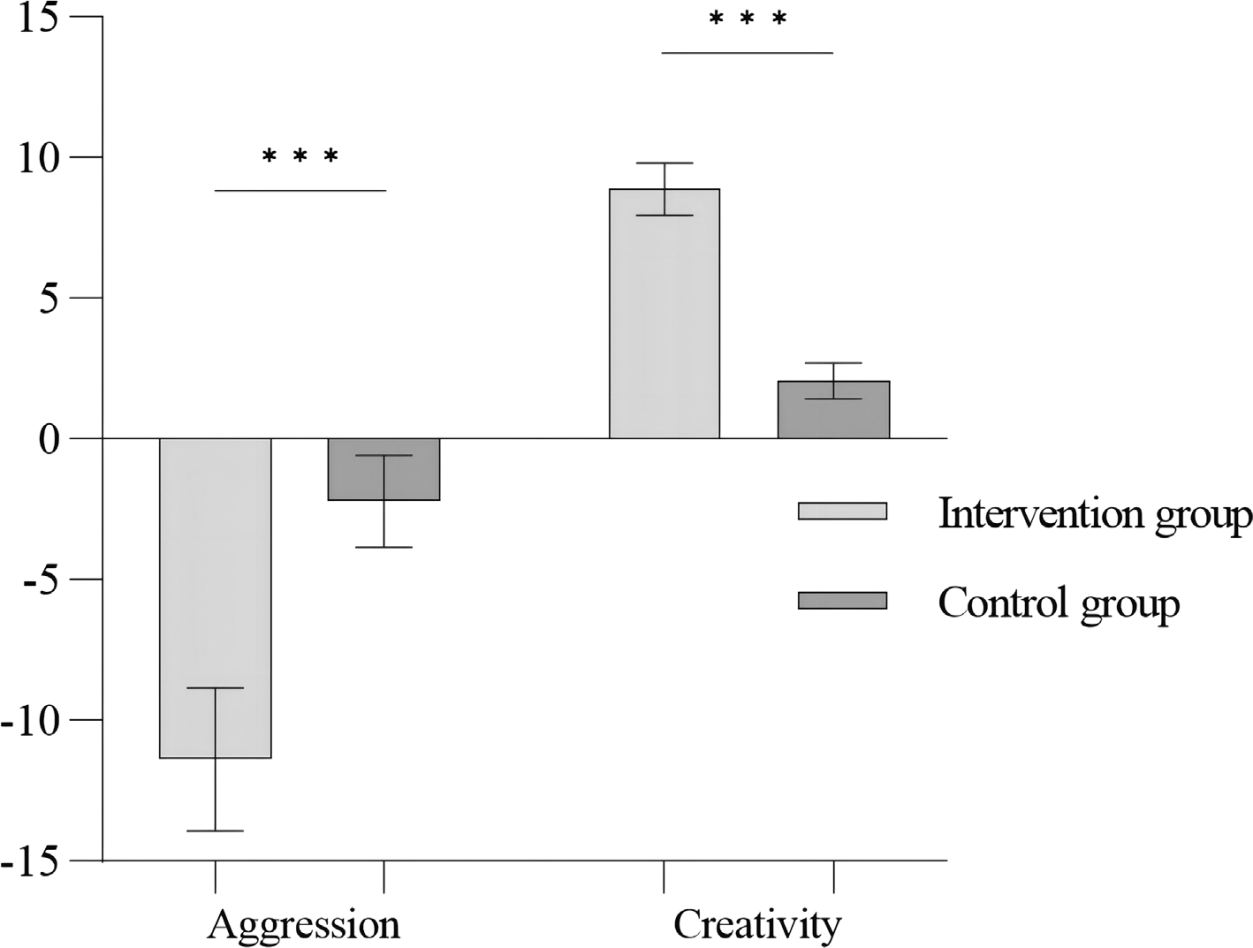
Group differences in the decrement in aggression and the increment in creativity.

Within‐subjects paired‐sample *t*‐tests were conducted for the control group. No significant difference was found between PreT (3.04 ± .28) and PoT1 (2.97 ± .36) scores on aggression (*t*_(29)_ = 1.34, *p* = .18, Cohen's *d* = 0.22) and subdimensions of aggression (*p*_all_ > .05). However, there was a significant difference (increase) between the PreT (10.43 ± 5.46) and PoT1 (12.50 ± 4.40) scores on creativity (*t*_(29)_ = −3.256, *p* = .03, Cohen's *d* = 0.412). This may be due to practice effects on the creativity test. Further analysis was conducted to compare the changes in aggression and creativity scores between the intervention and control groups before and after training (i.e., PoT1 – PreT). Independent‐sample *t*‐tests showed that the intervention group had a significantly larger decrease in aggression (*t*_(60)_ = 2.99, *p* < .001, Cohen's *d* = 0.49) and a significantly larger increase in creativity (*t*_(60)_ = 5.969, *p* < .001, Cohen's *d* = 1.26) than the control group (Figure 5).

### Correlation and regression analysis

Among the 172 participants, there was a significant positive correlation (*r* = .161, *p* < .05) between aggression (BPAQ) and creative trait (WCS) scores. This result suggests that there is a positive relationship between creativity and aggression and that individuals with high creativity and high aggression may share common personality traits.

Additionally, this study aimed to verify the hypothesis that CTT can transform aggression into creativity. We conducted a correlation analysis between the change (increase) in creativity and the change (decrease) in aggression (absolute value of the PoT‐PreT score) in both the intervention and control groups. The results showed that changes in creativity test scores were significantly correlated with changes in aggression in the intervention group (*r* = .404, *p* < .05), whereas there was no significant correlation between these two variables in the control group (*r* = −.067, *p* = .724). And only changes in hostility were significantly correlated with changes in creativity (*r* = .413, *p* < .05), whereas there was no significant correlation between other subdimensions of aggression (*rs* < .338, *ps* > .05). We also established a regression model for the intervention group, with the decrement in aggression as the dependent variable and the increment in creativity as the independent variable, that is, a decrease in aggression = 1.11 × increment in creativity +1.54. The regression coefficient of “increment in creativity” on “decrement in aggression” was significant, *t*(30) = 2.38, *p* < .05. These results suggest that an increase in creativity in the intervention group could predict a decrease in aggression, indicating that aggression can be transformed into creativity through the CTT program developed in this study.

### The long‐lasting effect of intervention

**FIGURE 6:**
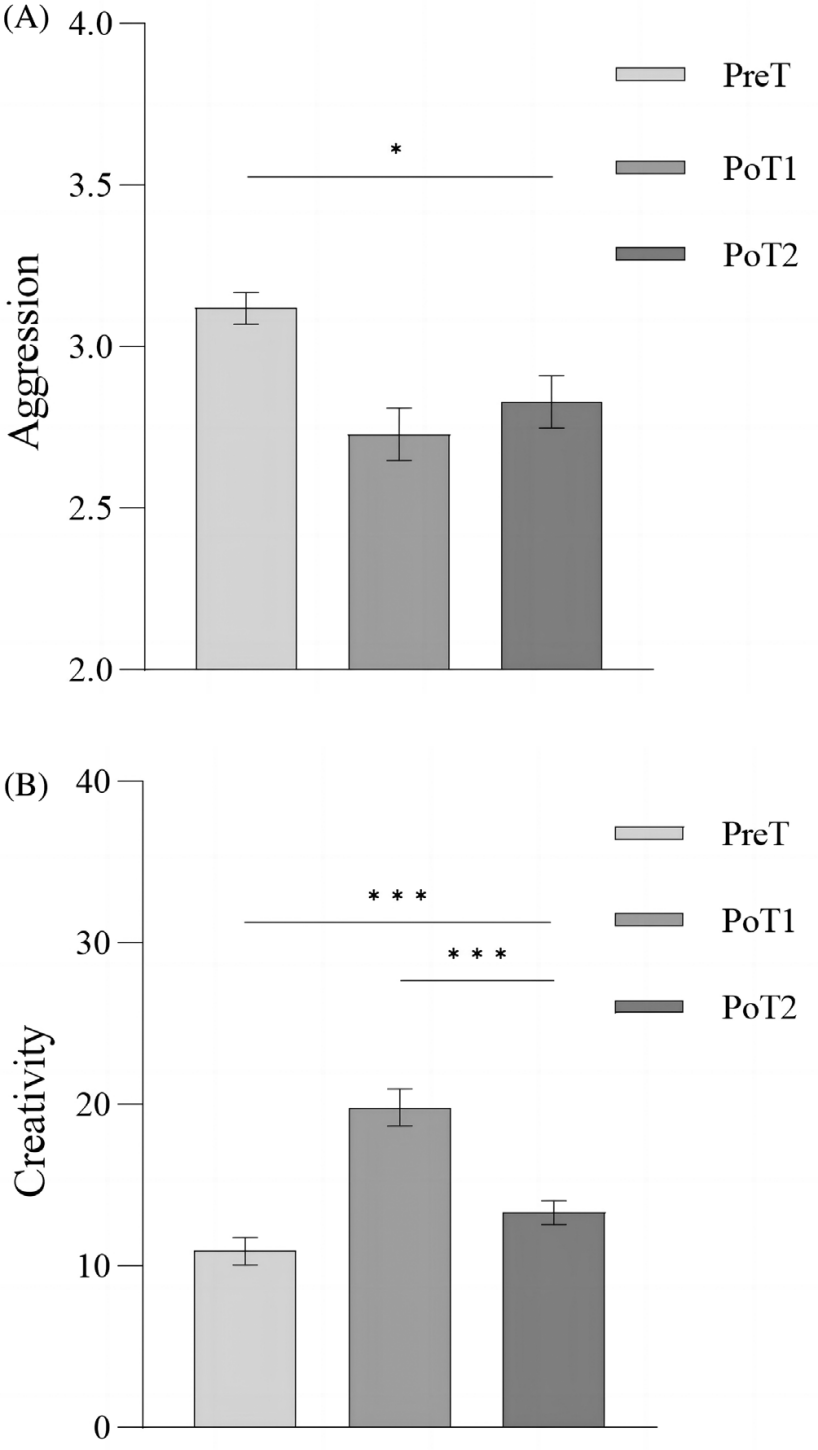
(A) Aggression at different points in time for the intervention group. (B) Creativity at different points in time for the intervention group.

To test whether the effects of the intervention project were long‐lasting, we conducted a repeated‐measures analysis of variance with test time points (PreT, PoT1, and PoT2) as the within‐subject factors and specific values of creativity or aggression levels in the intervention group as the dependent variables (Figure 6). There was a significant difference in aggression test scores, *F*(2,28) = 9.32, *p* < .001, η_p_^2^ = .391. *Bonferroni*‐corrected post‐hoc tests revealed no significant difference between PoT2 (2.83 ± .42) and PoT1 (2.73 ± .43) scores (*p* = .17), but both were significantly lower than PreT (3.12 ± .31) (*ps* < .05) (Figure 6A). There was also a significant difference in creativity test scores, *F*(2,28) = 42.15, *p* < .001, η_p_^2^ = .746. *Bonferroni*‐corrected post‐hoc tests revealed that PoT2 (13.32 ± 4.12) was significantly lower than PoT1 (19.82 ± 6.46) (*p* < .001) but was still significantly higher than PreT (10.94 ± 4.79) (*p* < .001) (Figure 6B). Additionally, we calculated the correlation between the increase in creativity scores and decrease in aggression scores from PreT to PoT2 in the intervention group and found a marginally significant correlation (*r* = .32, *p* = .07). These results indicate that the intervention project was still effective 6 months later, albeit with weakened effects.

## DISCUSSION

Based on empirical evidence showing an intrinsic relationship between aggression and creativity, and in particular, inspired by the effects of music intervention, which could simultaneously downregulate aggression and upregulate creativity (Pool & Odell‐Miller, 2011), this study designed and applied a CTT to a group of students with high aggression traits and tested CTT's efficacy in stimulating creativity and reducing aggression. The results of our study found that, regardless of whether compared with the control group or with the PreT before the intervention, four sessions of CTT could simultaneously increase creativity and reduce aggression, and this regulatory effect could last for at least 6 months. Moreover, we found that an increment in creativity could significantly predict a decrease in aggression, thus providing the first evidence for the CTT‐induced transformation of aggression into creativity.

First, more specifically, by measuring personality traits, our results confirmed Hypothesis 1, that there is a positive correlation between aggression and creativity, which is consistent with previous research results (Saggar et al., 2019; Tacher & Readdick, 2006). The intervention results demonstrated that after the application of CTT, the intervention group showed significant improvements in creativity and a decrease in aggression and subdimensions of aggression compared to the control group or the PreT before the CTT. This suggests that CTT for high‐aggression students can simultaneously enhance creativity and reduce aggression, thus confirming Hypothesis 2. Additionally, although each subdimension of aggression showed a significant decrease after the intervention, only physical aggression significantly differed in the between‐group comparison. This may suggest that CTT is more effective for intervening in aggressive overt behaviors. Previous research has linked rule‐breaking in aggression and mindset‐breaking in creative thinking (Petrou et al., 2020; Saggar et al., 2019) and suggested that they may share the same psychological mechanisms. This behavior is particularly common among rebellious, curious, and emotionally driven young individuals (Furnham & Bachtiar, 2008; Kashdan & Fincham, 2002), who are more impulsive and focus more on immediate problems (Bryan et al., 2023). Under these circumstances, CTT can redirect their impulsivity, and more importantly, provide a “vent” or a more constructive way for releasing internal tension of aggression and promoting creative problem‐solving.

Second, a medium effect size was observed throughout the intervention. This result may have been due to the selection of middle school students as participants. Specifically, during individual development, middle school students in adolescence have obvious impulsivity and sensitivity to the external environment and may exhibit a paradoxical phenomenon of prosocial and rebellious behaviors simultaneously because of their requirement for uniqueness and recognition from others (Blankenstein et al., 2020). In other words, this rebellious behavior can be reduced and expressed in a more acceptable manner if teenagers' needs are met. We inferred that CTT provides a stage for students to express their unique ideas and praise different perspectives, which may lead to a significant increase in creativity scores and, therefore, a medium effect size. In addition, because of the goal‐oriented nature of creativity in problem‐solving (Beaty et al., 2016) and thoughtful processes (Wallas, 1926), impulsivity may be constrained in the process. Group collaboration may promote the prosocial behavior of these more aggressive students and further amplify the intervention's effect on aggression; however, this needs further verification in future research.

Third, the intervention group demonstrated a significant positive correlation between the increase in creativity and decrease in aggression, supporting Hypothesis 3. Moreover, the degree of increase in creativity scores could significantly predict the degree of decrease in aggression scores, indicating that the enhancement of creative performance may reduce aggression levels, and suggesting the possibility that CTT may transform aggression into creativity. Within each subdimension of aggression, only changes in hostility were significantly related to changes in creativity. This is an interesting finding. We theorized that hostility is the key to determining whether the final product is aggressive or creative and may also explain why aggression is confused with exploration. Previous studies have reported mixed results regarding gifted students. Lau et al. (2004) studied highly gifted elementary school students and found that creativity was positively associated with social traits, such as sociability–leadership and social influence, as well as negative traits, such as aggressive–disruptive behavior and unpopularity. This implies that the disposition of highly creative individuals could be a mixture of contradictions containing both the pro‐ and antisocial dispositions (Gralewski, 2019) and a manipulation that could optimize the balance between these two dispositions would help transform aggressive and antisocial dispositions into creative and prosocial ones.

In essence, the transformation from aggression to creativity may require relinquishing narrow, self‐centered thinking (Anestis et al., 2009) and a destructive tendency to adopt a broader perspective and method to approach problems that consider the needs of others (Furnham & Bachtiar, 2008). Moreover, according to the broaden‐and‐build theory, CTT may teach more aggressive individuals to cope positively with their internal negative response bias to aggression, expand the limited scope of attention, establish enduring personal resources, and afford sufficient time for reflection on more nuanced solutions from broader, diverse, and constructive perspectives (Fredrickson, 2001). In addition to redirecting and enriching students' mental resources to facilitate improved and more creative adaptation to their environment, the CTT intervention program may also optimize students' executive control abilities (Nigg, 2017) to achieve a better balance between the top‐down, “cold” or rational‐driven way of thinking and the bottom‐up, “hot” or emotional‐driven way of thinking (Anderson & Bushman, 2002). Furthermore, it may also impart skills for appropriately generating, evaluating, and expressing new ideas, and encourage individuals to gain others' willingness of approval instead of excessively focusing on their own needs (Kasof, 1995).

Finally, after 6 months, the intervention effect in the experimental group showed a declining trend but remained significantly better than that for the PreT, partially supporting Hypothesis 4. This indicates that the effects of CTT could be partially maintained for at least 6 months. The marginally significant correlation between the increase in creativity and the decrease in aggression in the intervention group may have been influenced by some reversion caused by the long time interval. Considering the general view that regards the development of creativity as a long‐term continuous and accumulative process wherein the patterns between individuals, environments, and problem‐solving are dependent on the previous situation and serve as inputs for the next state (Kupers et al., 2019), it is reasonable to observe a decline in the intervention effect with time.

Compared to previous approaches to aggressive interventions, CTT appears to be a relatively novel method that offers distinct advantages. Generally, aggressive interventions for catharsis suggest that catharsis only works in the short term; however, if people use catharsis for a long time, it will increase aggression (Denzler & Förster, 2012). Beames et al. (2023) conducted a 12‐week self‐control training program and found that it did not reduce aggression, suggesting that self‐control training alone may not be sufficient to reduce aggression, and other means (such as cognitive reappraisal) are needed for intervention (Blake et al., 2018). Cognitive reappraisal requires individuals to suppress or disengage from emotional events (e.g., anger) and adopt a new perspective to re‐evaluate ideas to find a new solution (Clark, 2022). Denson (2015) suggested that cognitive reappraisal can reduce anger and aggression by putting negative emotions into an objective perspective and reinterpreting provocative behavior. Additionally, Barlett and Anderson (2011) found that reappraisal reduces aggressive behavior by reducing revenge and, in the process, meets specific goals. In other words, cognitive reappraisal may help individuals purposefully inhibit prepotent revenge ideation and divert attention toward generating constructive ideas to resolve conflicts (Perchtold‐Stefan et al., 2021). This is similar to the creative thinking process (Beaty et al., 2016). In particular, EEG research has found that when cognitive reappraisal is used to overcome negative emotions, the observed EEG activity exhibits a pattern similar to alpha power to conventional verbal creative ideation (Fink et al., 2017). It is conceivable that, in negative situations, narrow vision gradually opens up through cognitive reappraisal, resulting in more alternative solutions. In research on creative cognitive reappraisal, focused negative emotional cognition was transformed into new and favorable mental representations after active cognitive reappraisal (Wu et al., 2019). In this intervention, cognitive reappraisal likely bridges aggression and creativity. To form the final creative product, CTT may activate the process of cognitive reappraisal, which prevents the expression of aroused anger and immediate aggressive behaviors and thinking outside the box in the process of repeated thinking to achieve the goal of a more accepted solution to the problem. Moreover, the CTT did not directly take aggression as the target of regulation but instead tried to induce one's direction of thinking, problem‐solving approach, and way of expression more creatively and constructively, providing somewhat unexpected effects by reducing aggressive tendencies. Through positive creativity, adolescents gradually accumulate unique personal resources to help them achieve their identity and avoid identity diffusion (Sica et al., 2023). Currently, there is no evidence suggesting creativity training leads to certain adverse side‐effects. However, considering the possible dark side of creativity (Baas et al., 2019; Cropley et al., 2008; Gao et al., 2022; Hao et al., 2020; Lee & Dow, 2011) and its similar cognitive mechanisms in the constructive and destructive type of creativity (Baas et al., 2019; Gao et al., 2022; Harris & Reiter‐Palmon, 2015), adverse side‐effects from training are not entirely impossible. However, in terms of the CTT intervention used in this study, we found that people not only achieved a significant improvement in their creativity, but this improvement could also predict a decrease in aggressiveness, suggesting that the advantages of CTT could be double‐sided.

### Limitations and implications

This study has several limitations. First, the particularity of the sample selection may have led to a decrease in the experimental generalization effect. However, this study achieved significant results in the intervention of middle school students; whether the results can be extended to other age groups and regions requires further investigation. In addition, in the inter‐group comparison after the intervention, through post‐hoc analysis, we found that in the aggression, only a small power was found (1 − *β* = 0.656), which may be due to the small sample size of this study. More intervention data and more diverse groups need to be collected in future studies.

Second, the data were self‐reported, which may have weakened the objectivity of the assessments of aggression and creativity. Simultaneously, the test‐taking effect may have interfered with the results. Corresponding behavioral experiments can be carried out in the future, and the more objective Behavioral Assessment System for Children and Adolescents (Romero‐López et al., 2021) can be used to assess aggressive behavior or other measurement methods that are more ecologically valid in the context of specific situations (Love et al., 2023). In addition, computers serve as essential tools in the information age, and given the boom in research on video games and aggression (Blanco‐Herrera et al., 2019), future intervention studies can further incorporate computer‐based training methods (Benedek et al., 2006), integrating creativity training into daily life.

Finally, the hypothetical demonstration of the transformation of aggression into creativity requires further in‐depth exploration. The experimental results indicate that the intervention has an impact on both aggressive traits and creative behaviors. However, as aggression was not operationally manipulated in the experiment but rather assessed through a personality trait scale (Ridge et al., 2023), the significant positive correlation observed between increased creativity and decreased aggression scores does not definitively confirm the notion that aggression can be transformed into creativity. In addition, this study explored the possible role of cognitive reappraisal in the relationship between aggression and creativity. However, this remains a theoretical possibility, and further evidence from behavioral experiments and neuroimaging research can be conducted on the activity of brain regions such as the right hemisphere temporoparietal lobe, dorsolateral prefrontal cortex, posterior cingulate gyrus, and medial temporal gyrus (Fink et al., 2010; Raine et al., 2000; Sun et al., 2016; Wei et al., 2014) to support this hypothesis.

## CONCLUSION

From the perspectives of personality traits, psychological development, and cognitive mechanisms, there could be certain intrinsic links between aggression and creativity, which not only suggests the possibility that a single‐targeted approach to aggression intervention (e.g., inhibition) may produce a weak effect on aggression, but also the possibility of finding a way to promote creativity while reducing aggression. To accomplish this, the present study implemented a CTT intervention in a group of students with high aggression scores to examine whether it could simultaneously downregulate aggression and increase creativity. Our results showed that aggression decreased, and creativity increased significantly after the CTT intervention, and this regulatory effect could last for 6 months, although with a decline. In addition, the results showed that CTT‐induced creativity change (increase) significantly correlated with and positively predicted the CTT‐induced aggression decrease, thus suggesting that CTT could transform aggression into creativity.

## CONFLICT OF INTEREST STATEMENT

This work is original and has not been published elsewhere nor is it currently under consideration for publication elsewhere. The authors declare no conflicts of interest.

## ETHICS STATEMENT

The study was approved by the Ethics Committee of Capital Normal University.

## Data Availability

The data that support the findings of this study are available from the corresponding author upon reasonable request.
